# Use of traditional medicine for primary headache disorders in Kuwait

**DOI:** 10.1186/s10194-018-0950-3

**Published:** 2018-12-04

**Authors:** Jasem Y. Al-Hashel, Samar Farouk Ahmed, Fatemah J Alshawaf, Raed Alroughani

**Affiliations:** 1 Department of Neurology, Ibn Sina Hospital, P.O. Box 25427, Safat, 13115 Kuwait City, Kuwait; 2 Department of Medicine, Faculty of Medicine, Health Sciences Centre, Kuwait University, Kuwait City, Kuwait; 3 Department of Neurology and Psychiatry, Al-Minia University, Minia, Egypt; 4 Mubarak Al- Kabeer Hospital, Jabriya, Hawalli, Kuwait; 5 Division of Neurology, Amiri Hospital, P.O. Box 1661, Qurtoba, 73767 Kuwait City, Kuwait

**Keywords:** Migraine, Hijama, Tradional medicine, Kuwai

## Abstract

**Background:**

Traditional Medicine (TM) is widely accepted to be used for the treatment headache disorders in Kuwait however, researches remain poorly documented. We aimed to study the frequency of TM use and its impact in the primary headache patients.

**Methods:**

This is a cross sectional self-reported efficacy study, which was conducted in Headache clinic in Kuwait throughout 6 months. Patients who were diagnosed with primary headache disorders of both genders aged from 18 to 65 years were included. Self-reported questionnaires were distributed to patients who used TM in the previous year. It included demographic, and characteristics of headache (headache frequency, duration, number of analgesic used in days per month and severity of headache). TM queried included blood cupping (Hijama), head banding, herbal medicine (sabkha), and diet modification. It assessed characters of headache before and 3 months after the final TM session. Independent sample t test, paired sample t test and Chi-square test were used to compare between different values. *P* < 0.05 is considered significant.

**Results:**

A total of 279 patients were included. The mean age is 40.32 ± 11.75 years; females represented 79.6% of the cohort. Most patients (*n* = 195; 69.9%) reported the use of TM before presentation to headache clinic, mainly Hijama (47.3%). Cultural / religious beliefs were the cause of seeking TM in 51.3% versus 10% used it due to ineffective medical treatment and 8.6% used it because of intolerance of medical treatment. Patients used TM were older at the onset of headache (24.24 ± 10.67 versus 20.38 ± 8.47; *p* < 0.003), and had longer headache disease duration (19.26 ± 13.13 versus 16.12 ± 11.39; *p* < 0.044). All patients with chronic headache (100%) and most of episodic migraine patients (90.4%) sought TM while only (31.5%) of Tension type headache sought TM; *p* < 0.047. Patients who sought TM had more frequent episodes of headache, longer duration of attacks and higher number of days of analgesic-usage respectively over last 3 months before presentation to our side (9.66 ± 7.39 versus 4.14 ± 2.72; *p* < 0.001), (41.23 ± 27.76 versus 32.19 ± 23.29; *p* <. 0009), (8.23 + 7.70 versus 3.18 ± 3.06; *p* < 0.001). At 3 months after the final TM session, there was no significant reduction of frequency of headache days per month (9.19 ± 7.33 versus 8.99 ± 7.59; *p* < 0.50), days of analgesic use per month (7.45 ± 7.43 versus 6.77 ± 6.93; *p* < 0.09) and duration of headache (41.23 ± 27.76 versus 41.59 ± 27.69; *p* < 0.78). However, there was a significant reduction of the severity of headache (*p* < 0.02). Few patients (17.9%) reported adverse events with TM. Most of TM cohorts were not satisfied after receiving this type of medicine.

**Conclusion:**

TM was widely used in Kuwait for primary headache. Patients sought TM before seeking physician because they found them more congruent with their own cultural and religious beliefs. Health care professionals involved in the management of headache should be aware of this and monitor potential benefits or adverse events of TM. The usage of TM was not effective in reducing headache attacks and severity.

## Introduction

Primary headache ranked among the top three diseases contributors to the global burden of disease [1]. The effective treatment of headache disorders is still a moving field and a potential challenge to the neurologist [2] and the approach to its management reflects cultural diversity. Thus, many headache patients seek Traditional medicine (TM) for self-treatment and prevention of headaches. The socioeconomic development and literacy level of a community influences on how headache is perceived and medical treatment sought. Despite the availability of modern medicine, many people may rely more on traditional medical practice because of its cultural acceptability, easy accessibility, and affordability.

The World Health Organization (WHO) defined Traditional medicine (TM) as the sum total of knowledge, skills, and practices based on the theories, beliefs, and experiences indigenous to different cultures that are used to maintain health, as well as to prevent, diagnose, improve, or treat physical and mental illnesses [3]. WHO reported that 80% of the population of developing countries and 65% of the population of developed countries rely on TM for health care [4].

The reasons for people resorting to TM vary widely and include dissatisfaction with modern medicine [5], or congruency with users values and beliefs toward health and life [6]. Popular treatment based on the Qur’an and the Sunna of the Prophet.

Muhammad continues to be practiced in Muslim countries including Kuwait due to religious inspiration [7].

In Kuwait, folk beliefs may play a role in leading patients to try TM. Traditional medicine healers know Farry as an opening or a gap is the skull that would allow air to get inside the skull causing chronic headache. According to TM healers there are several methods to treat this disease. The Arabic word `hijama’ is often translated into English as `cupping’. Hijama is `blood cupping which is known as `wet cupping’, in which cups are placed on the surface of the skin, sucking the air out, and creating a vacuum to regulate the flow of blood and to stimulate life-energy, blood-cupping goes one step further, with the practitioner making small incisions on the surface of the skin in order to get rid the patient of blood stasis within the body. This blood is considered unhealthy blood in their beliefs [8]. Wet cupping is known to have also been practiced by many ancient cultures as in ancient Egyptians, India, Greeks, and Romans [8].

Other TM that are used in Kuwait for headache include Sabkha, head massage and diet modification. Sabkha (aka Labkha) is a herbal mixture that includes henna, prepared by specialized person and applied to the head and left for a few days [9]. Head banding or tying the head with a cloth to create pressure around the head to reduce the flow of blood to scalp can help to relieve the pain caused by swollen blood vessels. Application of an ice pack and local scalp pressure are the most commonly used non-pharmacological methods for temporary relief of migraine headache pain [10].

Physicians are often faced with patients who use or ask about TM. They are expected to guide the patient to provide information and give the best options for treatment, but often studies and data are not available to withdraw conclusions about such treatments. The efficacy and safety of many forms of TM are under research as compared to modern medicine [11].

Traditional healers often lack medical training and are not physicians and their limited medical knowledge may put patients at risk [12]. To our knowledge, no study to date has specifically investigated the use of TM in patients suffering from headache in Kuwait. The aim of our study was to assess the rates, reason and efficacy of TM use in patients with primary headache.

## Method

This A cross-sectional, questionnaire-based study was conducted in specialized headache clinic in tertiary hospital in Kuwait. Our study included patients aged 18–65 who are diagnosed with primary headaches confirmed by a Neurologist according to International Classification of Headache Disorders III (ICHD-III) [13], and onset of their headache preceded the use of TM for headache. To avoid recall bias, we included only those who reported use of TM for their headache in last year before presentation to headache clinic and completed three months after last TM session.

A questionnaire was distributed to the identified patients. Demographic data such as age, gender and education as well as the characteristics of the headache as number of years with headaches, headache frequency per month, duration of headache in hours, number of analgesics use days per month, headache pain intensity [14], and use of acute and prophylactic medication over last three month before presenting to headache clinic were collected. Headache pain intensity was measured on a four-point scale where 0 = no headache; 1 = mild headache; 2 = moderate headache; 3 = severe headache. This scale is recommended for use in research by the International Headache Society [14].

To avoid recall bias, we included only those who reported use of TM in last year before presentation to headache clinic and completed three months after last TM session. Also, we collected data of headache days, duration and number of analgesics used over the last three months before seeking clinical headache.

The self-reported efficacy questionnaire queried history of TM before presentation to headache clinic. It queried TM and its efficacy. The questions were: “Have you ever tried TM for headache?” “Which type of TM?” TM queried included blood cupping (Hijama), head banding, herbal medicine (sabkha), and diet modification which are known to be the most common used TM for headache in Kuwait. “Did you experience any efficacy in terms of reduction of headache frequency, duration, use of analgesics and/or intensity?” The questionnaire assessed characters of headache before and 3 months after the final TM session to assess the efficacy of TM. Participants were reported if they are satisfied or not satisfied with TM for headache. They were also asked to report any adverse events related to TM use.

### Statistical analysis

Statistical analyses were performed using SPSS 20.00 (SPSS Inc., Chicago, IL, USA). Simple descriptive statistical tests (mean and standard deviation) are used to describe the numerical values of the sample and the number and percentage of the non-numerical values. The significance of the differences between patients who used TM and others who did not used was determined using independent t test. Paired t-test was used to compare the frequency, duration of headache and number of analgesics use days, 3 months before and 3 months after the end of TM. A chi-squared test was used to compare between nonparametric variables. A probability of (P) ≤0.05 is accepted as significant.

All participants gave informed consents.

## Result

Out of 317 participants presented to headache clinic throughout the six months of study duration, 279 patients completed the questionnaires. The socio demographic characteristics of participants and characters of primary headache were outlined in Table 1. Most of them 79.6% were female. The mean age was 40.32 ± 11.75 and mean age at headache onset was 23.06 ± 10.19. In the cohort 44.4% had completed high school education.

**Table 1:** Comparison between patient who used TM and who did not use 3 months before presentation to headache clinic

Socio demographic data and Characters of primary headache	Total Sample (*n* = 279) Mean (SD)/No (%)	Patients Used TM (*n* = 194) Mean (SD)/No (%)	Patients did not use TM* (*n* = 85) Mean (SD)/No (%)	*P*
Mean Age	40.32 ± 11.75	40.42 ± 11.17	40.09 ± 11.75	0.83
Mean Age at onset	23.06 ± 10.19	24.24 + 10.67	20.38 ± 8.47	0.003*
Mean disease duration	17.08 ± 12.01	19.26 + 13.13	16.12 ± 11.39	0.044*
Gender				
Female	222 (79.6)	156 (70.3)	66 (29.7)	0.79
Male	75 (20.4)	39 (86.4)	18 (31.6)	
Education				
University	77 (27.6)	24 (28.6)	53 (27.2)	
High school	123 (44.1)	40 (47.6)	83 (42.6)	0.54
Primary school	79 (28.3)	20 (23.8)	59 (30.3)	
Diagnosis				
Episodic Migraine	125 (44.8)	113 (90.4)	12 (9.6)	
Tension Type	89 (31.9)	28 (31.5)	61 (68.5)	0.047*
Headache	35(12.5)	35 (100)	0	
Chronic Headache	30 (10.8)	19 (63.3)	11 (36.7)	
Others as TAC				
Frequency of headache/month	7.99 + 6.83	9.66 + 7.39	4.14 + 2.72	0.001*
Mean Duration of headache in hours	38.51 + 26.78	41.23 + 27.76	32.19 + 23.29	.0009*
Mean Number of analgesics/Month	7.71 + 7.04	8.23 + 7.70	3.18 + 3.06	0.001*
Severity of headache				
Mild	32(11.5)	0	32 (100)	
Moderate	165(59.1)	117 (70.9)	48 (29.1)	0.001*
Severe	82(29.4)	78 (95.1)	4 (4.9)	
Used treatment for headache	76 (27.2)	72 (36.9)	4 (4.8)	
No drugs	72 (25.8	48 (24.6)	24 (28.6)	0.001*
Prophylactic treatment	131 (47)	75 (38.5)	56 (66.7)	
Symptomatic treatment				

*TM*: Traditional Medicine

*TAC*: Trigeminal autonomic cephalgia

Episodic migraine was the most presented headache in our cohort 44.8% followed by tension-type headache 31.9%, chronic migraine 12.5%, and other types of primary headache disorders 10.8% including cluster headache, paroxysmal hemicranias and other trigeminal autonomic cephalgias.

Most of our patients 69.5% tried TM in the last year before presentation to headache clinic. Table 1 compare the Socio demographic data and Characters of primary headache in those who used TM and those who do not use it over last three month before presentation to headache clinic to avoid recall bias. No significant differences were found in seeking TM depending on gender (*p* < 0.79). However, those who sought TM are significantly older at onset of headache (*p* < 0.003) and have longer disease duration (*p* < 0.044). There was no statistically significant difference between education status and use of TM (*p* < 0.54), (Table 1).

All participants with chronic headache, 90.4% participants with episodic migraine versus only 31.5% of Tension Type Headache (TTH) Tried TM (*p* < 0.047), (Table 1). Those who used TM when presented to headache clinic had significant frequent headache attacks (*p* < 0.001), longer duration of headache attacks (*p* < 0.009) and more frequent use of analgesic compared to those who did not use TM. The use of TM was significantly higher among those who do not use medical treatment compared to those who used it (p < 0.001). (Table 1).

The frequency, reasons and satisfaction of TM were shown in Table 2. Most of the participants used Hijama 65.6% either alone 47.3% or with Sobakh 18.3%, followed with those who were treated with Sobkh and few of our cohort used other modalities of TM as head banding, head massage and diet modifications. Culture/religion beliefs were the cause of seeking TM in 51.3% versus 10% used it due to ineffective medical treatment and 8.6% used it because of intolerance/fear of medical treatment. Most of participants 69.9% used TM before seeking neurologist. Only 26.2% reported that they are satisfied with TM. Few of them 17.9% reported adverse events as allergy, pain or trauma.

**Table 2:** Analysis of frequency, reasons and satisfaction of TM (*N* = 194)

Variables	Number (%)	*P* value
TM		
Cupping (Hijama)	132 (47.3)	
Sobakh	81(29.1)	0.001*
Cupping (Hijama) and Sobakh	51 (18.3)	
Others (Head banding, head massage, special diet)	33 (11.8)	
Cause of asking for TM		
Cultural/ Religious	143 (51.3)	0.001*
Ineffective medicine Intolerance to/fear of medicine	28 (10) 24 (8.6)	
Time of TM		
Before seeking Neurologist	105 (69.9)	0.001*
After seeking Neurologist	90 (30.1)	
Satisfaction		
Satisfied	51 (26.2)	.001*
Not satisfied	144 (73.8)	
Adverse events		
Yes	35 (17.94)	0.001*
No	160 (82.05)	

*TM*: Traditional Medicine

Self-reported efficacy of TM was outlined in Table 3. At 3 months after the final TM session, there was no significant reduction of frequency of headache days per month (*P* < 0.50), days of analgesic use per month (*P* < 0.09) and duration of headache (*P* < 0.78). However, there is significant reduction of the severity of headache for 3 months (*P* < 0.02).

**Table 3:** self-reported efficacy of TM (N = 195)

Variables	Before TM use	3 month after TM use	P
Frequency of headache/month	9.66 ± 7.39	9.44 ± 7.70	0.47
Mean Duration of headache in hours	41.23 ± 27.76	41.59 ± 27.69	0.78
Mean Number of analgesics days /Month	8.04 ± 7.62	7.34 ± 7.20	0.09
Severity of headache			
Moderate	117(60)	144(73.8)	
Severe	78 (40)	51 (22.2)	0.03*

*TM*: Traditional Medicine

## Discussion

TM is widely used for primary headache disorder in Kuwait, however efficacy has not been proven. We aimed to highlight the frequent use of traditional medicine in our community. TM, is sometimes used instead of conventional medicine and may lead to delay the diagnosis and prober management. We did not study its efficacy or its procedures. We included the patients who finished the course of TM according to their traditional healers. To us Hijama, head banding, sabkha, and diet modification all are non-conventional medicine that interfere with proper management in our community. Evidence indicated that traditional medicine was not only used for the healthcare of the poor; its prevalence increased in countries where allopathic medicine is predominant in the healthcare system [3]. The prevalence rate of TM use in Gulf is high. It is 67% in a United Arab Emirates [15] and 42% in Saudi Arabia [12]. To our knowledge, this is the first study of TM use by headache patients in Kuwait. We reported that 69.5% of patients who attended headache clinics used TM. When comparing our result with other western studies of non-conventional medicine use our figure of 69.5% headache clinic patients using TM is higher than the 31%, 40% and 29% shown in Italian migraine, chronic tension type headache and cluster headache patients respectively [16–18], 32% of headache clinic in United Kingdom [19] 44.4% [20], 41.3% [21] in United state of America but less than the 81—85%reported in Austrian and German [22] and United state of America [23] headache clinic patients. This might reflect cultural and regional differences on how and by whom complementary and alternative medicine therapies were provided.

In our study, TM users were older than TM non-users. There was no significant difference between both groups regarding gender or education level. It was surprising that highly educated subjects used TM which is of unknown mechanism of action. They preferred the tradional medicine because of its spiritual origin.

In the last three months before presentation to headache clinic, were more likely to suffer from more frequent attacks, more intense headaches for a longer period of time, when compared to non-TM used; which is consistent with previous studies [17, 19, 22, 24, 25]. Worse headache may be the cause of seeking TM since more than 50% of our participants sought TM because of their religion beliefs. We think that the headache get worse in TM users who did not received the required adequate conventional medicine when compare to TM non-users. We noticed also that all chronic headache patients who were presented to headache clinic tried TM and TM users have significant long disease duration.

This study reported that 70% of participants used TM before seeking help from neurologist which is the reverse of previous western studies, around 2/3 cases in headache clinic in Italy [16, 17], 67% [19] in headache clinic in UK and 62% of general population in UK [26] sought conventional treatment before non-conventional medicine. The majority of participants in western studies gave the reason for using non-conventional medicine that they believed it would effectively treat headache _after ineffective conventional treatment. However most of the participants in our study, 69.5% used TM before conventional treatment because it is in congruent with their culture and religion believes. TM healers are usually trusted members of the community [27].

Hijama was the most common used TM for primary headache, in 65.6% of our cohort. The religious roots of hijama is that the Prophet Muhammad advocated its practice. It is taken from Prophetic tradition. The prophet Mohammed (peace be upon him) referred to hijamah for curing an illness [28]. It is noted in al-Buhary and Muslim, the two most authoritative Sunni compilations of the Prophet’s sayings, that Muhammad reportedly said that healing is “in the incision of a cupper” [29]. Prophet mohammed didn’t specify which disease hijama will heal, moreover, he didn’t stop people from seeking medical advice at first, so doing it has some blessing since following a practice of the prophet but in view of advanced medicine the prophet didn’t stop any person from seeking a doctor. However, few of our patients tried TM because they were not satisfied by efficacy of conventional fear of or intolerance to side effects.

TM use has the potential to be harmful if patients use it with non-educated personnel (especially when using instruments with poor hygiene resulting in infections) or if they stop effective conventional therapies while using a TM therapy. Physicians need to be understanding, supportive and open minded when interacting with patients use TM. Healthcare providers should educate patients about their TM use, monitor potential benefits or adverse events and educate patients about conventional medicine. Physicians and TM practitioners need better communication and coordination of care in order to provide the best available patient benefit. TM unfortunately used by some unprofessional healers just to gain money which is totally misuse of the healthcare system and using the illness of the people solely for financial reasoning. Also, some of those healers they don’t know how to deal with vasovagal attacks or loss of consciousness that some patients can have especially during hijama.

Although the self-reported efficacy of TM is modest, the use of TM is high in this study. Conventional medicine may not always improve the headache, and some patients do not tolerate acute and/or prophylactic medicine due to side effects or contraindications. Similar to other studies [16, 17] some patients may wish to avoid medication due to possible side effects or risk for medication tolerance.

### Limitations of the study

The study includes patients attending clinic who are a special subset of primary headache sufferers. They may have refractory or disabling headaches, so the results of this study might not reflect the majority headache patients.

The efficacy of TM are based on self-reports and therefore subjected to recall bias. We tried to minimize recall bias by including the patients who used TM only in the last year before enrolment in the study and collecting data headache days, duration and number of analgesics used over the last three months before seeking clinical headache.

Personal causality, individual perception and understanding of pain in addition to belief in TM modalities could affect the subjective judgment.

of headache relief and satisfaction of TM.

## Conclusion

The frequent use of TM for primary headache in our community is a major concern. Health care professionals involved in the management of headache patients should be aware of this. There is a need for evaluation of the benefits and safety of TM therapies for headache. Community awareness for medical headache treatment should be improved. Healthcare providers should educate the patients about TM use, monitor potential benefits or adverse events. Most of our patients, at some point, seek some or all traditional medicine because of their cultural and religious believes. Conventional healthcare providers and TM practitioners need better communication and coordination of care in order to provide the best available patient care and safety. Those healers should have also some sort of license in order to practice TM this may protect safety of the patients.

For future research, it will be interesting to conduct a general population survey to see if rates of TM use in treating headache are similar to those attending headache clinics. TM may be helpful as a complementary medicine so we need to study its efficacy and safety in details and we recommend to coordinate with traditional healers to avoid hazards of TM and keep this type of treatment under our observation.

## Abbreviations

CH: Chronic headache
TAC: Trigeminal autonomic cephalgia
TM: Trational medicine
TTH: Tension type headache
